# Unravelling novel microbial players in the breast tissue of TNBC patients: a meta-analytic perspective

**DOI:** 10.1038/s41522-025-00816-5

**Published:** 2025-09-09

**Authors:** Hannah H. Rashwan, Nour H. Marzouk, Rana A. Youness, Mohamed El-Hadidi, Raghda Ramadan, Mohamed Mysara

**Affiliations:** 1 Bioinformatics Group, Centre for Informatics Science (CIS), School of Information Technology and Computer Science (ITCS), Nile University, Giza, Egypt; 2 Biology and Biochemistry Department, Faculty of Biotechnology, German International University, New Administrative Capital, Egypt; 3 Cancer and Genomic Sciences, School of Medical Sciences, College of Medicine and Health (CME), University of Birmingham Dubai, Dubai, United Arab Emirates, Dubai, United Arab Emirates; 4 School of Biotechnology, Nile University, Giza, Egypt

**Keywords:** Metagenomics, Microbiome

## Abstract

Triple-negative breast cancer (TNBC) is the most aggressive subtype of breast cancer (BC), accounting for nearly 40% of BC-related deaths. Emerging evidence suggests that the breast tissue microbiome harbors distinct microbial communities; however, the microbiome specific to TNBC remains largely unexplored. This study presents the first comprehensive meta-analysis of the TNBC tissue microbiome, consolidating 16S rRNA amplicon sequencing data from 200 BC samples across four independent cohorts. Our analysis highlights the enrichment of Azospirillum genus as well as butyrate-producing species, namely *Gemmiger formicilis* and *Anaerobutyricum soehngenii*, potentially influencing TNBC aggressiveness and clinical outcomes. Additionally, our functional analyses reveal the involvement of the TNBC microbiome in several pathways associated with chronic inflammation, increased cellular proliferation, invasion, and metastasis. This study uncovers novel microbial players in TNBC that could explain its aggressiveness and poor prognosis, and warrants further investigation into microbiome-driven interventions.

## Introduction

Triple-negative breast cancer (TNBC) represents a particularly aggressive subtype of breast cancer (BC), characterized by the absence of estrogen receptor (ER), progesterone receptor (PR), and human epidermal growth factor receptor-2 (HER2)^1^. Despite accounting for only 10–15% of all BC cases, TNBC is the most aggressive BC subtype where it contributes to 40% of BC-related mortalities and exhibits high rates of metastasis and relapse within five years post-treatment^2–4^. Compared to the other subtypes, TNBC has the worst prognosis with limited treatment options due to the lack of hormone receptors^5^. Given the absence of effective targeted therapies for TNBC, it is crucial to investigate the biological underpinnings of its aggressiveness and poor prognosis. Taking into consideration that therapies directed at specific molecular markers have rarely succeeded^6^, there is a pressing need to investigate alternative, non-traditional targets to better explain and address the abnormal behavior of TNBC.

During the past decade, studies revealed a complex microbial ecosystem within breast tissue—once thought to be sterile—indicating microbial involvement in BC pathogenesis through mechanisms such as microbial translocation^7^. Further research has begun to characterize the difference in microbiome between cancerous and healthy breast tissues. These studies highlight how microbes contribute to cancer progression through immune modulation, direct carcinogenic activities, and affecting cellular pathways involved in cell proliferation or apoptosis^8^. Some studies also suggested that tumour-resident microbiome play an important role in promoting BC metastasis^9^. Consequently, identifying these specific microbial players or their metabolic products could aid in enhancing BC patient outcomes.

Current studies on the BC microbiome do not focus on unveiling the uniqueness of TNBC compared to the other subtypes, overlooking the distinct microbial signatures that may be specific to TNBC^10^. Recently, a primary study by Wang et al. delved into the microbial landscape of TNBC, identifying microbial species linked to cancer response in TNBC in addition to uncovering notable differences in specific metabolites^11^. To contrast these findings more comprehensively, a meta-analysis is needed to consolidate findings from various studies and accurately identify key microbial factors that could contribute to the progression of TNBC.

In this work, we conduct the first meta-analysis characterizing the tissue microbiome of TNBC compared to non-TNBC, aiming to pinpoint the exact microbial species influencing TNBC pathogenesis. For this purpose, we incorporate samples from the complete pool of primary studies on BC with a clear distinction of TNBC from the other subtypes. Thus, we utilize our standardized methodology that integrates unprocessed raw data from all studies - referred to as a mega-analysis - to ensure comparability and effectively address inter-study heterogeneity. This meticulous approach not only elucidates these differences but also aims to uncover novel microbial targets, potentially explaining the aggressiveness and poor prognosis of TNBC.

## Results

### Systematic search results

**Fig. 1:**
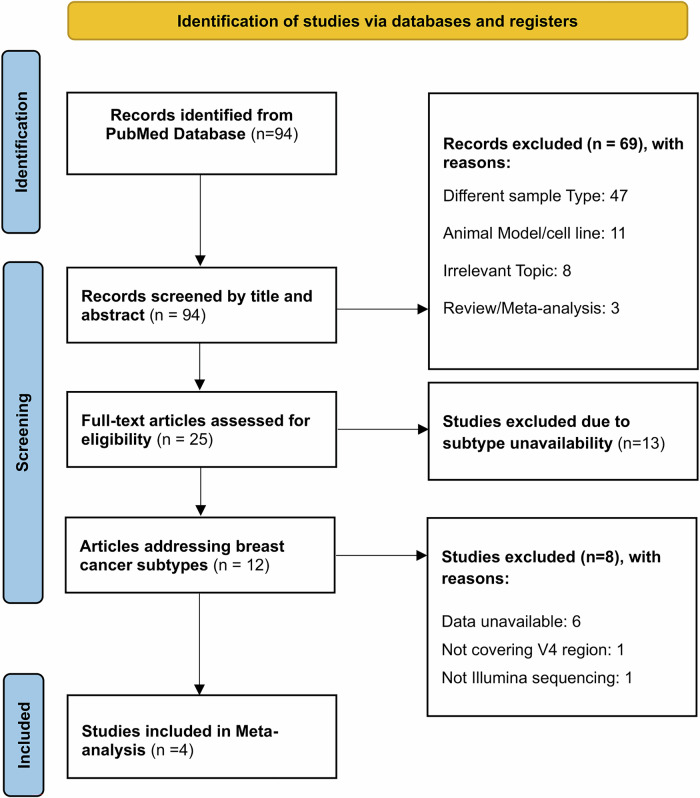
PRISMA chart for meta-analysis. The chart illustrates the selection process for studies included in the meta-analysis, where four homogenous studies were finally retained.

A comprehensive search of the PubMed database yielded a total of 94 records. Initial screening based on titles and abstracts led to the exclusion of 69 entries for various reasons (Fig. 1). Further filtration during the full-text review resulted in the exclusion of 13 articles because they did not address the BC subtypes. Of the remaining 12 studies, six were further excluded due to data unavailability. Additionally, one study was excluded because it did not use Illumina sequencing technology, and another was excluded for not covering the variable region 4 (V4). Ultimately, the meta-analysis included four homogeneous studies that met all the inclusion criteria and were relevant to the BC subtypes under investigation. All systematic search results with reasons for inclusion and exclusion are available in Supplementary Table 1.

This meta-analysis combines four studies that explore the breast tumour microbiome across different regions and technologies (Table 1). Studies A and B, both from the United States, share similarities in their focus on racial differences in breast microbiomes and utilize Illumina Miseq technology. Study A examines the V3-V4 region, while Study B focuses on the V4 region and emphasizes differences in microbial communities across race, stage, and tumour subtype. Study C, conducted in China, also investigates breast tumour microbiomes but incorporates the V4 region and utilizes the more advanced Illumina Novaseq 6000 technology. Finally, Study D from Italy examines microbiome dysbiosis in breast tumours, analysing the V4-V6 region with Illumina MiSeq technology. The common factors among these studies include the use of breast tissue samples, with most employing the V4 region and Illumina MiSeq sequencing. Based on the IQC metric, which evaluates OTU pair correlations across studies, our analysis revealed high homogeneity among studies (Supplementary Fig. 1).

**Table 1:** Studies included in the meta-analysis

Study	Country	Publication date	TNBC samples No.	non-TNBC samples No.	V. region	Sequencing Technology	Accession No.	Reference
A	United States	2020	46	20	V3-V4	Illumina Miseq	PRJNA637875	^93^
B	United States	2019	15	45	V4	Illumina Miseq	Requested by Email	^94^
C	China	2022	10	40	V4	Illumina Novaseq 6000	PRJNA842933	^95^
D	Italy	2022	3	20	V4-V6	Illumina Miseq	PRJNA759366	^96^

The table presents an overview of research studies examining the tissue microbiome of breast cancer. *Triple Negative Breast Cancer (TNBC)*

### Distinctive microbial composition and decreased diversity in TNBC compared to non-TNBC

**Fig. 2:**
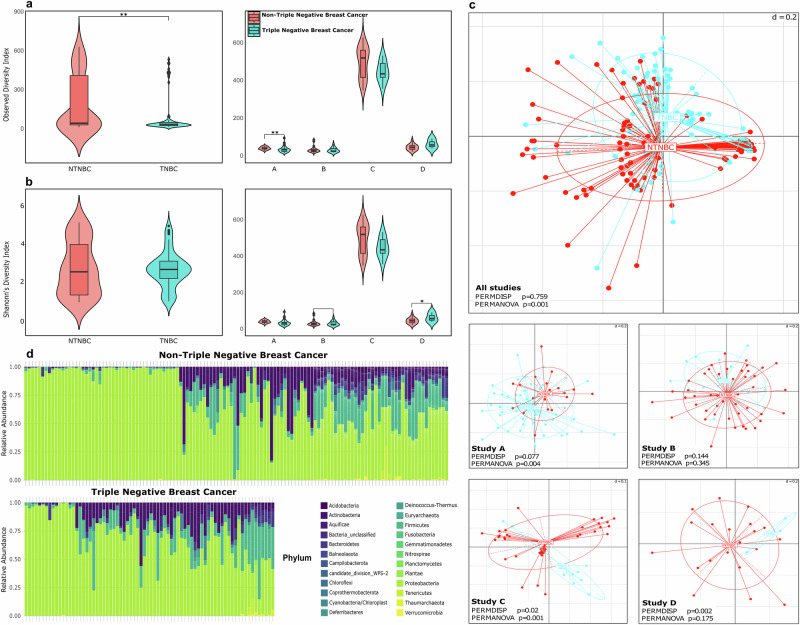
Comparative analysis of microbial diversity and composition in TNBC and non-TNBC samples. **a** Observed Diversity Index, quantifies species diversity across classes and within each study. **b** Shannon’s Diversity Index illustrates the estimated richness of microbial species across classes and within each study. **c** Beta diversity compares microbial community composition between TNBC and non-TNBC across all studies and individually per study. **d** Bar plots illustrate the relative abundance of microbial phyla in TNBC and non-TNBC samples.

In comparison to non-TNBC tissues, our exhaustive analysis of the TNBC tissue microbiome included evaluations of alpha diversity, beta diversity, and microbial composition, all of which were obtained from the four independent studies. The observed index of alpha diversity revealed a markedly reduced microbial diversity in TNBC tissues relative to their non-TNBC counterparts (*P* < 0.05; MW; Fig. 2a) Upon closer inspection of the diversity indices for the four studies, only study A showed substantial difference in the alpha diversity (*P* < 0.05; MW; Fig. 2a). In contrast, the Shannon diversity index exhibited only a slight, non-significant decline at the class level, with Study D being the sole exception where significant results were observed (*P* = 0.03; MW; Fig. 2b).

To assess the beta diversity, dimensionality reduction was employed in the Rhea pipeline on all studies combined using unweighted UniFrac distances; however, the study effect overshadowed any possible difference between the TNBC and non-TNBC samples. When examining the differences within each study independently, clusters between the two classes—especially in studies A and C—arose (*P* < 0.05; PERMANOVA, Fig. 2c). In addition, we employed the RCM - a more comprehensive approach -to all the studies combined, which considers compositionality and takes the study effect into account to clarify any distinctions between the two classes (*P* < 0.05; PERMANOVA; Supplementary Fig. 1). Lastly, bar plots were employed to depict the phylum-level microbial composition within TNBC and non-TNBC tissues. These visual representations highlighted a notable reduction in microbial density in TNBC samples when compared to their non-TNBC counterparts. Specifically, the phyla Bacteroidetes and Firmicutes were significantly less abundant in TNBC tissues. This distinction underscores potential differences in the microbial communities associated with these BC subtypes, suggesting a possible link between microbial composition and cancer phenotype (Fig. 2d).

### Butyrate-producing bacteria as potential biomarkers and carcinogenic pathways in the TNBC tissue microbiome

**Fig. 3:**
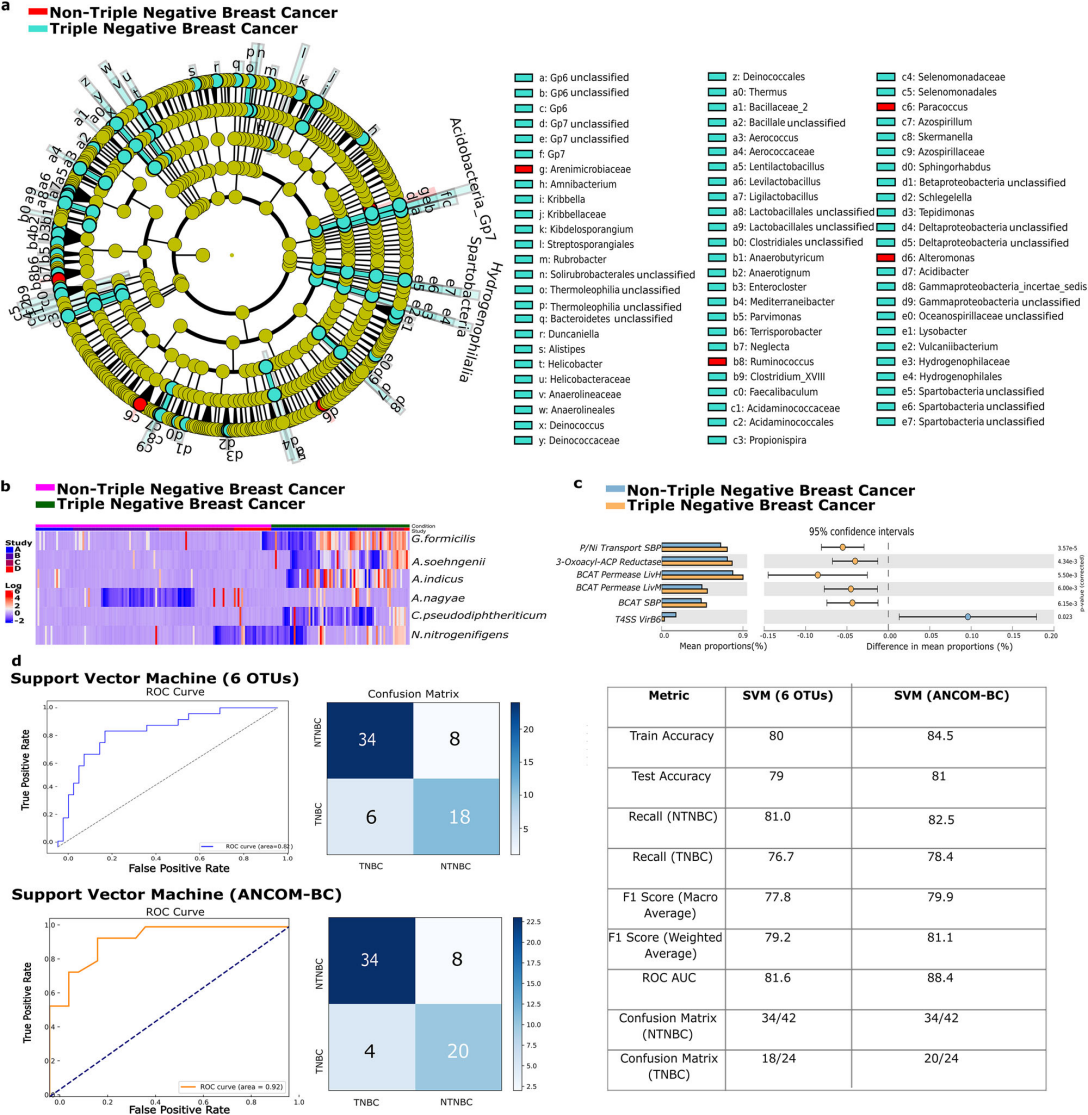
Detailed visualization of bacterial significance and predictive modelling in TNBC and non-TNBC. **a** A cladogram that highlights significant bacteria at various taxonomic levels, provides a hierarchical view of microbial differences. **b** A heatmap displays the significant bacterial species differentially abundant between TNBC and non-TNBC. **c** Results from STAMP, identify key functional pathways impacted in TNBC. **d** Confusion matrices and Receiver Operating Characteristic (ROC) curves for machine learning models, demonstrate the predictive accuracy and validity of these models in distinguishing between TNBC and non-TNBC samples.

In order to differentiate between TNBC and non-TNBC samples at various distinct taxonomic levels, we implemented a strict criterion where we intersected the results of ANCOM-BC and *MetaDE* in MetaOmics. ANCOM-BC is particularly adept at handling the compositional nature of microbiome data, accounting for potential biases that traditional differential abundance methods might overlook. *MetaDE*, on the other hand, aggregates data across multiple studies, enhancing the statistical power and reliability of the findings through combined effect sizes. We identified the observed changes in microbial abundance, with 164 species found to be significantly enriched in TNBC samples (FDR < 0.05; ANCOM-BC). The results were visualized in a cladogram where many taxa were significantly enriched in TNBC samples across several taxonomic levels. (Fig. 3a). The analysis revealed a consistent enrichment of several taxa in TNBC, including the order Hydrogenophilales and Lactobacillales together with several families including Hydrogenophilaceae, and Azospirillaceae. At the genus level, Anaerobutyricum, Azospirillum, and Skermanella were among the genera significantly enriched in the TNBC samples (Supplementary Table 2). With in-depth analysis, we were able to identify the differentially abundant species that differentiate TNBC from non-TNBC. Three species were found to be significantly upregulated in TNBC compared to both non-TNBC samples and HC samples (FDR < 0.05, Supplementary Fig. 4) according to both ANCOM-BC and *MetaDE* analyses namely *Gemmiger formicilis, Anaerobutyricum soehngenii*, and *Acinetobacter indicus* while *Anaerococcus nagyae, Corynebacterium pseudodiphtheriticum*, and *Novosphingobium nitrogenifigens* were found to be downregulated (Fig. 3b). Further assessment of the upregulated species revealed that TNBC consistently exhibited distinct microbial signatures relative to each non-TNBC subtype (Supplementary Fig. 3). To validate these findings, a subgroup analysis excluding Study D was performed, which confirmed the robustness and consistency of the microbial community structure (Supplementary Fig. 2).

Our functional analysis, conducted using STAMP, identified five Kegg Orthologies (KOs) that were significantly enriched in TNBC. These include the branched-chain amino acid transport system (BCAT) ATP-binding protein and the (BCAT) permease protein, both of which are involved in ABC transporters and quorum sensing. In addition, 3-oxoacyl-ACP reductase was found to be involved in several pathways including fatty acid biosynthesis, prodigiosin biosynthesis, biotin metabolism, and fatty acid metabolism (Fig. 3c). Further association analysis using MicrobiomeAnalyst highlighted substantial differences in several pathways between TNBC and non-TNBC. Notably, pathways such as Steroid biosynthesis, Arginine biosynthesis, Cysteine and methionine metabolism, Amino sugar and nucleotide sugar metabolism, and Phenylalanine, tyrosine, and tryptophan biosynthesis were significantly enriched in TNBC (FDR < 0.05, detailed list available in Supplementary Table 3).

### Machine learning

In this analysis, we developed and evaluated two support vector machine (SVM) classifiers of varying complexity to distinguish between TNBC and non-TNBC cases. To ensure a consistent and unbiased evaluation framework, Stratified K-Fold Cross-Validation (k = 3) was employed, preserving the class distribution across all folds and minimizing sampling bias. To address the inherent class imbalance in the dataset, we applied a weighting strategy that automatically adjusted class weights based on their frequency. This approach assigned greater importance to the minority class (TNBC), thereby reducing bias toward the majority class (non-TNBC).

The first model was trained on six OTUs identified as significant by both ANCOM-BC and MetaDE. It achieved a mean training accuracy of 80.0% and a mean test accuracy of 79.0%. The recall for non-TNBC cases was 81.0%, while TNBC cases achieved a recall of 76.7%. The F1 score reflected balanced performance, with a macro average of 77.8% and a weighted average of 79.2%. Additionally, the model demonstrated competent discriminatory capacity, with a ROC-AUC score of 81.6%. According to the confusion matrix, the model correctly classified 34 out of 42 non-TNBC cases and 18 out of 24 TNBC cases (Fig. 3d).

The second model, trained on all OTUs identified by ANCOM-BC, incorporated a correlation-based feature selection step. Features with absolute correlation values exceeding 0.175 were retained, resulting in a final input set comprising OTUs from Study A, Study C, Study D, and 21 microbial species (Supplementary Note 1). This model showed improved performance, achieving an average training accuracy of 84.5% and a test accuracy of 81.0%. Recall rates were 82.5% for non-TNBC and 78.0% for TNBC cases. The F1 score improved as well, with a macro average of 79.9% and a weighted average of 81.1%. The ROC-AUC score increased to 88.4%, indicating stronger discriminatory power. The confusion matrix revealed that the model correctly identified 34 out of 42 non-TNBC cases and 20 out of 24 TNBC cases (Fig. 3d).

## Discussion

TNBC is recognized as the most aggressive form of BC, characterized by a unique tumour microenvironment (TME) that contributes significantly to its poor prognosis^12–15^. Despite extensive investigations aimed at elucidating the mechanisms underlying this aggressiveness, these efforts have been largely inconclusive^6^. Recent advancements in understanding the interplay between the microbiome and cancer pathogenesis have shed light on the role of intra-tumour microbes that are known to provoke inflammatory and immune responses, which in turn contribute to tumour progression and patient outcomes^16–18^.

Several studies have characterized the BC tissue microbiome, yet it was not until the work of Wang et al. that the microbial composition of TNBC compared to the other subtypes was assessed^11^. Thus, it was pressing to contrast these findings with the vast pool of previous studies to properly unravel the exact microbial players implicated with TNBC and their potential role in the TME. In this work, a comprehensive systematic search for all studies characterizing BC subtypes and tissue microbiome was conducted. Among a pool of twelve studies providing subtype-specific information, four primary analyses with available, homogeneous data were retrieved. This meta-analysis is the first to focus on the microbial composition unique to TNBC, identifying three novel bacterial species potentially pivotal in contributing to the subtype’s aggressiveness and poor prognosis.

Our analysis demonstrated a lower alpha diversity and a distinct microbial composition of TNBC compared to non-TNBC, in line with the findings of Wang et al.^11^. Particularly, we reported elevated levels of butyrate-producing bacteria, namely *Gemmiger formicilis* and *Anaerobutyricum soehngenii*, within TNBC tissues compared to both non-TNBC samples -with their different subtypes- as well as HCs^19,20^. *G. formicilis* has been previously detected in the gut microbiome of TNBC patients, which suggests potential microbial translocation from the gut to the tumour tissue^21^. Additionally, this bacterium has been linked to colitis, highlighting its potential proinflammatory role in TNBC^22^. *A. soehngenii*, which was also found abundant in TNBC tissues, has been previously reported to be more enriched in other types of cancer such as colorectal cancer (CRC), underscoring its significance in gastrointestinal malignancies and potential implication in TNBC^20,23^.

The role of butyrate as a tumour suppressor has been widely investigated, yet several studies highlight the “Butyrate Paradox,” where butyrate exhibits contradictory effects in-vivo and in-vitro, depending on concentration, context, and timing of exposure^24–33^. Specifically, lower concentrations (in the micromolar range) of butyrate can stimulate cellular proliferation, whereas higher concentrations (in the millimolar range) tend to inhibit it^34,35^. For instance, low doses of butyrate (0.05 mM) exerted mitogenic effects and promoted hepatocyte proliferation, while higher doses (5 mM) were found to be cytotoxic to a murine hepatocyte cell line^30^. Similarly, subtherapeutic doses of sodium butyrate (NaB) have been associated with increased cell migration in ER + BC cells^36^.

At therapeutic levels, NaB demonstrates potent anti-tumour effects across several BC subtypes. It induces apoptosis and G2/M cell cycle arrest through caspase-10 activation, oxidative stress, and epigenetic reprogramming^37^. In TNBC cell lines such as BT-20, MDA-MB-231, MDA-MB-453, and MDA-MB-468, NaB has shown anti-proliferative effects, including cell cycle arrest, apoptosis, and downregulation of mutant p53^38–41^. For instance, NaB induces reactive oxygen species (ROS) production, caspase activation, and mitochondrial membrane disruption in the MDA-MB-468 cell line, leading to apoptosis^39^. It is worth noting that although MDA-MB-231 cells undergo apoptosis and growth arrest, they require higher NaB concentrations compared to MCF-7 ER+ cells^40^. NaB’s cytotoxicity is dose-dependent, with significant effects observed between 10–30 mM, and approximately 50% viability reduction at 30 mM; MDA-MB-231 cells begin to respond from 3 mM^42^. Butyrate was also found to inhibit epithelial-mesenchymal transition (EMT) and invasiveness in TNBC by restoring E-cadherin expression, blocking β-catenin nuclear translocation, and suppressing MEK/ERK signalling^43^.

Emerging evidence suggests butyrate may also exhibit tumour-promoting effects under certain conditions. TNBC cells (e.g., MDA-MB-231) exhibit relative resistance to NaB-induced cytotoxicity compared to other non-TNBC cell lines (MCF7 & T-47D), particularly at concentrations of 2–10 mM^44^. Interestingly, NaB has been shown to enhance G6PDH activity in these cells, upregulating the pentose phosphate pathway and boosting antioxidant defences, features commonly associated with metastatic potential^44^. Additionally, opportunistic colonizers like *Fusobacterium nucleatum*, a butyrate-producing bacterium, are enriched in late-stage BC tumours, where they may promote immune evasion and metastasis^45,46^.

Findings from other malignancies also support the Butyrate Paradox, highlighting butyrate’s dual role. In lung cancer, low-dose butyrate enhances tumour growth and metastasis through extracellular matrix remodelling, upregulation of MMP15, and HDAC2-dependent activation of the oncogenic lncRNA H19, which also promotes M2 macrophage polarization^47–50^. In hepatocellular carcinoma, NaB concentration below 0.5 mM increases proliferation and, with chronic exposure, promotes hepatocyte proliferation and liver fibrosis^28,30^. In CRC, low butyrate concentrations contribute to a pro-inflammatory microenvironment, encourage colonization by SCFA-producing microbes, and promote both proliferation and chemoresistance^32,51,52^.

**Fig. 4:**
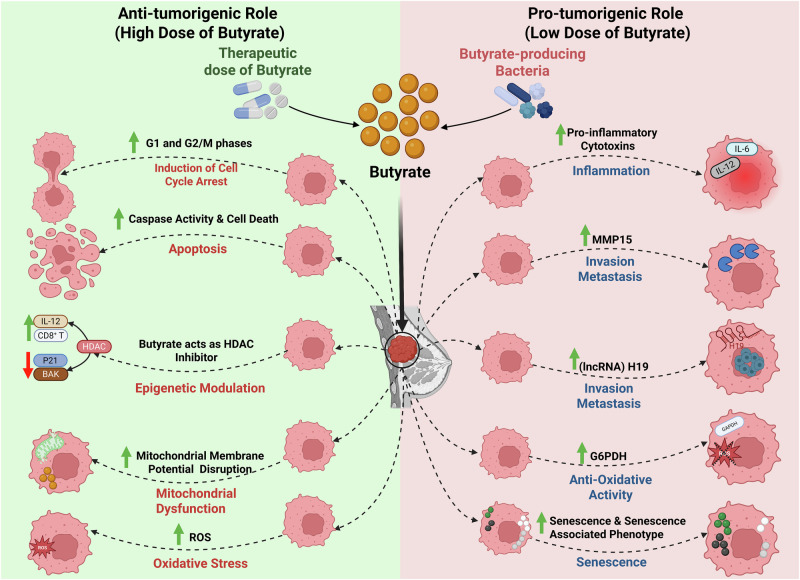
Dose-dependent anti-tumorigenic and pro-tumorigenic hypothesized roles of butyrate in TNBC. The diagram illustrates butyrate’s hypothesized dual role in TNBC: high doses (left) exert anti-tumour effects through epigenetic modulation and immune activation, while low doses (right) may promote tumour growth, metastasis, and immune evasion in specific microbial environment contexts. HDAC: Histone Deacetylase, P21: Cyclin-dependent kinase inhibitor 1 A, BAK: Bcl-2 homologous antagonist/killer, MMP15: Matrix Metalloproteinase 15, ROS: Reactive Oxygen Species, lncRNA H19: Long non-coding RNA H19, G6PDH: Glucose-6-phosphate dehydrogenase.

Interestingly, a recent study identified two Porphyromonas species enriched in CRC patients, which accelerate tumorigenesis by inducing cellular senescence via butyrate secretion. Invasion by these bacteria was observed in CRC tissues, accompanied by elevated butyrate levels and senescence-associated inflammatory phenotype. Notably, tumorigenesis was abolished in ApcΔ14/+ mice when bacterial butyrate-synthesis genes were disrupted—strongly suggesting a causal role for microbial butyrate in promoting CRC development^53^. Similarly, in prostate cancer, butyrate enhances proliferation via IGF-1 signalling^54^. Collectively, these findings emphasize that butyrate’s effects are highly dependent on concentration, exposure duration, tissue type, and microbiome context. We hypothesize that such mechanisms may contribute to the aggressive behaviour of TNBC and present a proposed model of these dose-dependent effects (Fig. 4), though further in vivo validation is warranted.

Another interesting finding from our analysis is the enrichment of the *Azospirillaceae* family and *Azospirillum* genus—particularly *Azospirillum oryzae*—in TNBC tissues, as identified by ANCOM-BC. While the enrichment of the *Azospirillum* genus has been previously reported in the BC microbiome^55^, to our knowledge, this is the first study to report the presence of *A. oryzae* specifically in TNBC tissue compared to non-TNBC cases. Notably, Wang et al. documented a positive correlation between *Azospirillum* and LysoPE, specifically LysoPE (20:4), a metabolite significantly elevated in highly metastatic TNBC cells^11,56^. This relationship may be mechanistically linked to the enzymatic activity of *Azospirillum* species, including *A. oryzae*, which possess a patatin-like phospholipase (PL) enzyme (A0A6N1AJL0; PL-A2 domain) capable of cleaving host phosphatidylethanolamine (PE) to produce LysoPE. In turn, LysoPE is known to activate the LPA₁/Gi–PLC–IP₃–Ca²⁺ signalling cascade and modulate downstream MAPK pathways (ERK, JNK, p38), thereby promoting proliferation and migration in TNBC cells^57^. Furthermore, the genus has been positively associated with tumour-infiltrating lymphocytes (TILs) in TNBC, implicating a possible role in modulating the immune microenvironment^11^. While experimental validation is needed, these findings suggest that *Azospirillum*, particularly *A. oryzae*, may influence TNBC progression through both metabolic and immunological pathways.

Our functional and association analyses of the TNBC tissue microbiome elucidated significant pathways contributing to various cancer hallmarks in TNBC. Here we report the enrichment of steroid biosynthesis, biotin metabolism, and amino sugar and nucleotide sugar metabolism pathways, aligning with the observations of Wang et al.^11^. They reported elevated levels of 17-β-estradiol-2,3 quinone in TNBC, suggesting a unique estrogen disposition that may contribute to the oncogenesis of this subtype^58,59^. Our analysis also detected notable differences in the pathways linked to amino acids and fatty acids synthesis in TNBC samples compared to other subtypes, aligning with another study on BC subtypes^60^. Furthermore, ABC transporters were enriched in our results where they have been implicated in tumour development, metastasis, angiogenesis, and the epithelial-mesenchymal transition in BC^61–64^. Specifically, in TNBC, ABCC11 has been linked to increased aggressiveness^61^, and elevated levels of ABCB1 are associated with metastatic spread^65^. Moreover, the overexpression of ABC transporter proteins is known to mediate drug resistance in TNBC^66^. It is also worth mentioning that quorum sensing was significantly enriched in our analysis, highlighting its role in inducing cell invasion and promoting angiogenesis as reported in other studies on BC^67^.

While this meta-analysis offers novel insights into TNBC-associated microbial signatures, it is important to recognize several limitations. The cohort size, particularly in Study D, was small but addressed via random-effects models and subgroup analyses confirming result consistency. Although the technical variability (e.g., 16S rRNA regions) was minimized through standardized preprocessing, residual primer bias may persist. Geographic variability was handled by incorporating study location as a confounder and validating cross-study consistency using IQC metrics. Heterogeneity in non-TNBC groups limited subtype-specific comparisons, though TNBC-specific microbial features were robust in subgroup analyses. Butyrate-producing taxa (e.g., *Gemmiger formicilis, Anaerobutyricum soehngenii*) were enriched, but metabolic activity was inferred, not measured—highlighting the need for experimental validation via metabolomics profiling and in vitro/in vivo models’ assays. Additionally, gnotobiotic mouse models colonized with defined microbial communities will help validate the causal role of microbial metabolites in tumour progression.

In conclusion, our study offers novel insights into the intricate relationship between the TME, the microbiome, and their combined impact on TNBC progression. Our primary objective was to comprehensively characterize the tissue microbiome of TNBC to elucidate its influence on disease progression and patient outcomes. This study breaks new ground by reporting, for the first time, the abundances of *Gemmiger formicilis, Anaerobutyricum soehngenii, and Azospirillum oryzae* elucidating their potential roles in influencing TNBC aggressiveness and prognosis. This meta-analysis is the first to elucidate the differences between TNBC and non-TNBC which aims to uncover novel microbial targets, potentially explaining the aggressiveness and poor prognosis of TNBC. Future studies should confirm these findings through experimental validation and the potential need for microbiome-based therapies.

## Methods

### Systematic search

On July 1st, 2023, a comprehensive search was conducted in PubMed database, using specific keywords such as “Breast Cancer,” OR “Breast Tissue,” OR “Breast Tumour, AND “16S”, AND “Microbiome,” OR “Microbiota.”, which yielded a total of 94 results. Thorough manual screening resulted in finding 12 studies that specifically included the BC subtypes. The inclusion criteria required samples originating from a tissue microbiome, case-control or cohort studies regardless of their geographic location, and studies that use the 16S rRNA sequencing method and Illumina sequencers to cover the V4 or V3-V4 hypervariable regions. As for the exclusion criteria, studies without publicly accessible data or metadata were excluded, as were any studies that mentioned antibiotic administration within three months prior to sample collection. This study was reported in accordance with the PRISMA guidelines. The completed PRISMA checklist is provided in Supplementary Table 4.

### 16S data pre-processing

Raw 16S rRNA amplicon sequencing data were collected from four distinct studies, resulting in a comprehensive dataset of 302 samples. Studies were retrieved from the following accession numbers PRJNA637875, PRJNA842933, and PRJNA759366 except for one study that was retrieved by email from the corresponding author. The studies’ heterogeneity was assessed using the Internal Quality Control (IQC) metric, which evaluates OTU pair correlations across studies. To minimize inconsistencies due to different preprocessing pipelines and analytical strategies across studies, all raw reads were re-analysed using a uniform bioinformatics pipeline (OCToPUS v.1.0^68^), following a mega-analysis approach. This standardized reprocessing ensured consistency in quality control, read trimming, denoising, alignment, chimera removal, and OTU clustering. Study A included 66 samples, all of which were retained for analysis. In contrast, Study B initially consisted of 91 samples; however, 11 were excluded due to having an unknown origin. Study C, which originally contained 94 samples, had 29 samples excluded due to patient exposure to chemotherapy, leaving 65 samples for inclusion. Lastly, Study D started with 68 samples, but 11 were discarded due to their classification as unknown BC subtypes. Consequently, the final dataset for analysis comprised 268 samples, including 68 healthy controls (HCs), which were removed. A total of 200 BC samples were thus subjected to downstream analysis.

To mitigate technical biases related to sequenced region variability, we computationally aligned all reads to the SILVA reference database V4 region (positions 13,862–22,588) (v.138.2^69^). This region-specific normalization allowed for consistent comparisons across studies despite variation in original primer sets (e.g., V3–V4 vs. V4–V6). The k-mer frequency approach was employed to individually de-noise the forward and reverse raw readings using SPAdes (v.3.5.0^70^). Contigs were generated by combining the de-noised paired-end reads with Mothur (v.1.39.1^71^) and base ambiguity-containing contigs were removed. After the remaining contigs were aligned to the SILVA database (v.138.2^69^) targeting the V4 region, contigs that either did not align, exceeded eight homopolymers, or had peculiar lengths were eliminated. The trimmed sequence alignment was further refined using the Illumina Paired-End Denoiser (IPED) algorithm (v.1.0^72^) and CATCh (v.1.0^73^) was employed for de novo chimera removal. OTU clustering was conducted at a 97% identity level using UPARSE (implemented in USEARCH v.8.1.186^74^). Subsequently, the default criterion of 80% was used to conduct taxonomic classification using the Ribosomal Database Project (RDP) dataset (v.19^75^).

To further mitigate biases related to sequencing depth and compositionality, we applied a multi-faceted statistical approach. ANCOM-BC (Analysis of Composition of Microbiomes with Bias Correction) corrected for compositional effects while controlling FDR^76^; Random Effects Models accounted for study-level heterogeneity^77^; and Residuals and Covariance Modelling (RCM) used log-ratio transformations to reduce library size dependency^78^. Together, these strategies ensured robust and reproducible cross-study comparisons.

### Diversity analysis

Two indices were employed to quantify alpha diversity: the observed diversity index and the Shannon diversity index^79,80^. The observed diversity index measures species richness, including rare species, to estimate a community’s total species count. In contrast, the Shannon Diversity Index evaluates species richness and evenness, offering a comprehensive view of biodiversity. In order to compare the two groups, we implemented the Mann–Whitney *U* test, a non-parametric approach, applying FDR < 0.05^81^.

The microbial communities of various samples were compared using beta diversity analysis. First, the analysis employed the Rhea pipeline (v.1.1^54^), which employs a distance matrix based on the unweighted UniFrac distances. This approach considers the phylogenetic relationships and the presence/absence of taxa, which enabled the visualization of beta diversity via NMDS. In addition, beta diversity was further analysed using RCM while taking study effect into account to account for compositionality^78,82^. The significance of the differences between groups was evaluated using PERMANOVA (Permutational Multivariate Analysis of Variance) with FDR < 0.05 for both NMDS and RCM^83^.

### Biomarkers detection through meta-analysis

The ANCOM-BC method was employed as it addresses the compositionality and sampling biases that are inherent in microbiome datasets^76^. Taxa were considered significantly differentially abundant if their FDR < 0.05. We also used a cladogram to demonstrate the hierarchical relationships and relative abundances of taxa with significant differential abundance^84^.

Furthermore, the *MetaDE* module of MetaOmics was employed to integrate the individual results from each of the four studies in order to identify the significantly differentially expressed OTUs^85^. A meta-analysis, as well as a subgroup analysis (excluding study D), were performed to compare the tissue microbiome of TNBC samples with that of non-TNBC where we employed the REM, assuming a random effect size across studies^77^. Moreover, an additional analysis comparing TNBC samples to individual non-TNBC subtypes (excluding Study A due to inconsistent classifications) was performed. The effect sizes were combined to generate a summary effect size and confidence interval within the REM framework, utilizing appropriate statistical methodologies. Subsequently, differentially expressed OTUs were identified by selecting OTUs with FDR < 0.05 based on both ANCOM-BC and *MetaDE* results in line with the methodology employed by Marzouk et al.^86^. Lastly, the NCBI Nucleotide BLAST was implemented to classify the identified bacteria with complete coverage^75^. A targeted analysis was conducted incorporating HC samples from Studies B, C, and D (as Study A lacked HC samples). Focusing on the key TNBC-associated OTUs, comparative effect size plots were generated.

### Functional prediction

Metabolic and functional capabilities of the microbial communities in TNBC and non-TNBC samples were inferred through functional prediction. This analysis employed *themetagenomics* package and the ‘*t4f*‘ function in R^87^. Using the STAMP (Statistical Analysis of Metagenomic Profiles) software, the predicted functional profiles were analysed statistically and visualized^88^. The analysis utilized Welch’s *t*-test (two-sided) with ratio of proportions to identify significant functional differences. Utilizing the Kyoto Encyclopaedia of Genes and Genomes (KEGG) database, we conducted a search for applicable metabolic pathways, and association analysis was performed using MicrobiomeAnalyst for pathway elucidation^89,90^.

### Machine learning

The data underwent a standardized pre-processing protocol to ensure uniformity and optimize downstream machine learning performance. Two support vector machine (SVM) classifiers were developed. The first model targeted a refined feature set comprising microbial species identified as significant by both ANCOM-BC and MetaDE. The second model included a broader set of features, encompassing all operational taxonomic units (OTUs) identified by ANCOM-BC. Feature selection was applied exclusively to the second model using a correlation-based filtering approach, whereby features with an absolute correlation greater than 0.175 were retained.

For both models, the dataset was randomly partitioned into training (80%) and testing (20%) subsets, using a fixed random state of 50 to ensure reproducibility and optimal performance. To mitigate class imbalance and improve model robustness, Stratified K-Fold Cross-Validation (k = 3) was employed, preserving the class distribution across all folds and minimizing sampling bias during training and evaluation^91^. Furthermore, both SVM classifiers were configured to automatically balance class weights, assigning greater importance to the minority class (TNBC) and reducing bias toward the majority class (non-TNBC)^92^. Model performance was assessed using multiple evaluation metrics, including F1-score, receiver operating characteristic (ROC) curves, confusion matrix, recall, and overall accuracy.

## Supplementary information

Supplementary Data

## Data Availability

All data generated or analysed during this study are included in this published article and its supplementary information files. Studies were retrieved from the following accession numbers PRJNA637875, PRJNA842933, and PRJNA759366 except for one study that was retrieved by email from the corresponding author.

## Code availability

The underlying code for this study is not publicly available but may be made available to qualified researchers on reasonable request from the corresponding author.
