# Supplementary material for: Unravelling novel microbial players in the breast tissue of TNBC patients: a meta-analytic perspective
Source: NPJ Biofilms Microbiomes. 2025 Sep 9;11:182. doi: 10.1038/s41522-025-00816-5 (PMC12420793; doi:10.1038/s41522-025-00816-5)
Supplement: Supplementary file 1 — Supplementary Data [file 41522_2025_816_MOESM1_ESM.pdf]

Supplementary Materials

Unravelling Novel Microbial Players in the Breast Tissue of TNBC Patients: A Meta-analytic Perspective

Supplementary Figure 1: Study Effects on RCM Plots and Internal Quality Control (IQC) Metrics Across Datasets

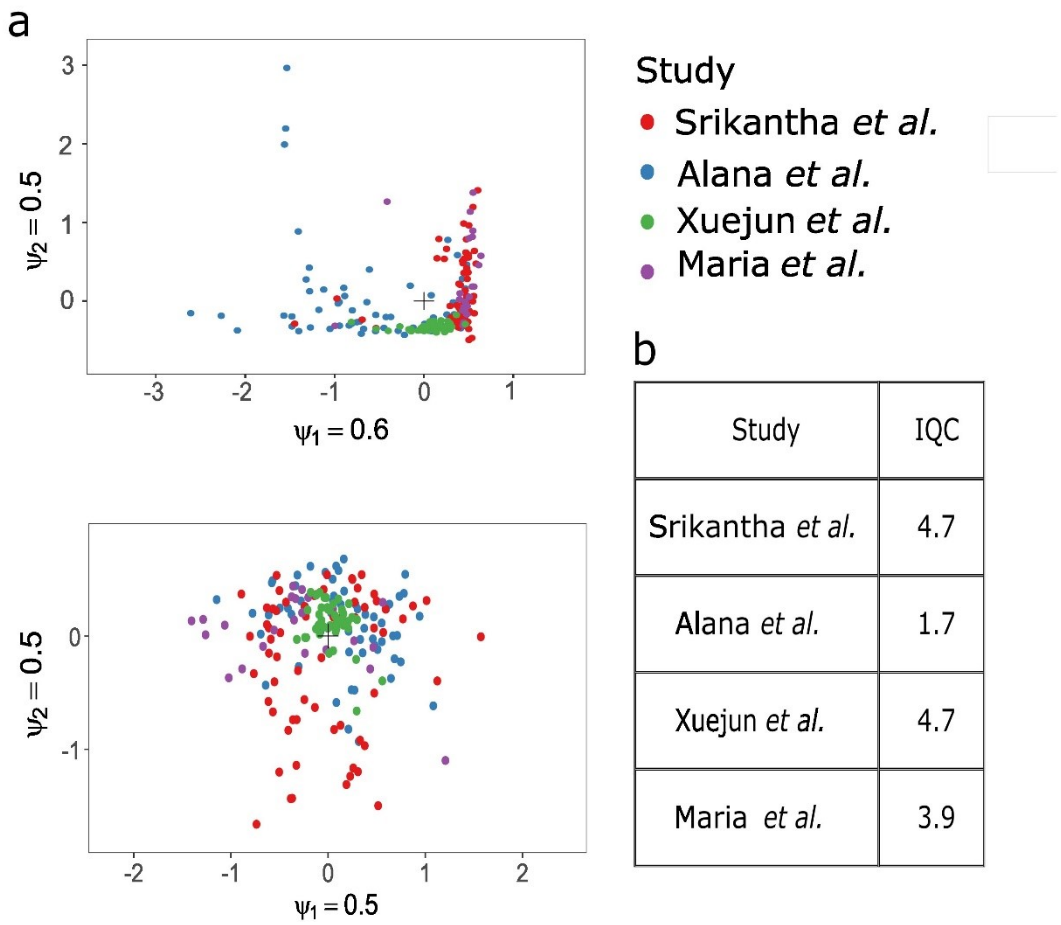

Study	IQC
Srikantha *et al.*	4.7
Alana *et al.*	1.7
Xuejun *et al.*	4.7
Maria *et al.*	3.9

a) RCM plots before and after accounting for the study. B) Quality control results for each of the studies using Internal Quality Control (IQC) metric, which evaluates OTU pair correlations across studies.

Supplementary Figure 2: Overlap of OTUs Between Main (ABCD) and Subgroup (ABC) Analyses

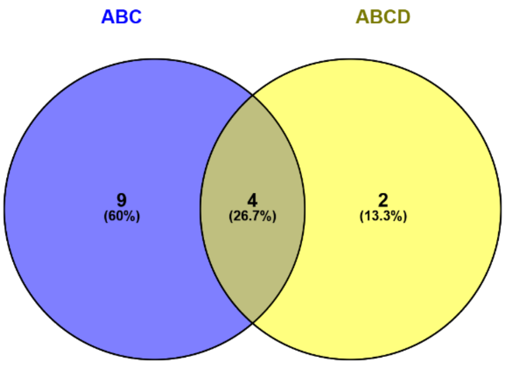

Venn diagram illustrating common OTUs between the main analysis including studies A, B, C and D, and the subgroup analysis including studies A, B and C only.

Supplementary Figure 3: Subtype-Specific Abundance Profiles and Sample Distribution Across Studies

a

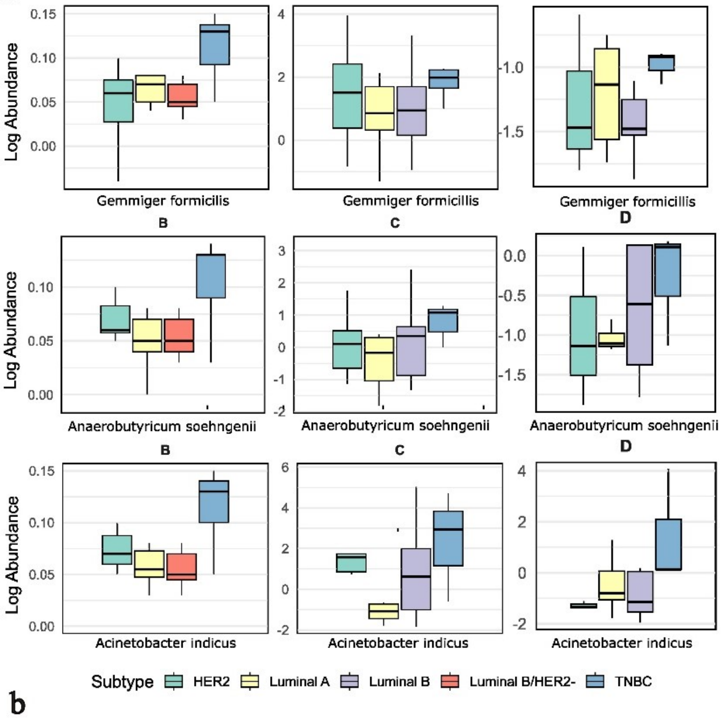

b

Group/Study	A	B	C	D
HC	0	19	15	34
Luminal A	0	24	17	10
TNBC	46	15	15	3
HER2	0	10	11	3
TPBC	20	0	0	0
Luminal B	0	0	32	7
Luminal B/HER2	0	12	0	0

a) *Log abundances of TNBC and Non-TNBC subtypes for Gemmiger formicilis, Anaerobutyricum soehengenii and Acinetobacter indicus. b) Contingency table for the NTNBC samples.*

Supplementary Figure 4: Forest Plot of Odds Ratios for Differentially Abundant Microbial Species in TNBC vs. Controls

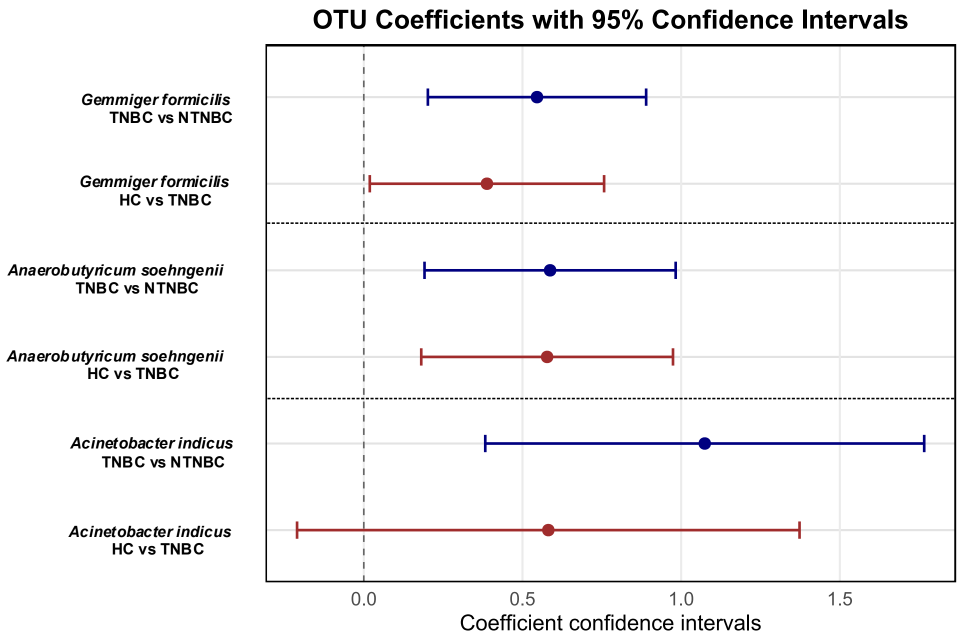

Forest plot showing odds ratios (OR) and 95% confidence intervals (CI) for differentially abundant microbial species in TNBC versus non-TNBC and healthy controls (HCs). Blue markers represent species enriched in TNBC, while red markers represent species enriched in HCs compared to TNBC. Error bars represent 95% confidence intervals.

Supplementary Note 1 : Selected features for ANCOM-BC model

Photobacterium damselae, Hyphomonas jannaschiana, Sphingomonas panaciterrae, Ammoniphilus resinae, Clostridium paraputrificum, Thomasclavelia spiroformis, Alteromonas taga, Vibrio gigantis, Vulcaniibacterium thermophilum, Prevotella disiens, Desnuesiella massiliensis, Elongatibacter sediminis, Neglectibacter caecimuris, Brachymonas denitrificans, Chakrabartyella piscis, Glaciihabitans tibetensis, Azospirillum oryzae, Parasynechococcus marenigrum, Stenotrophomonas nematodicola, Leucobacter viscericola, Vitiosangium cumulatum, Amnibacterium setariae, Dongia rigui, Parvimonas micra, Ruegeria conchae, Ligilactobacillus araffinosus, Marivivens niveibacter, Fusobacterium varium, Mucilaginibacter gilvus, and *Bacteroides acidifaciens*.

Supplementary Table 1: Systematic Search Results and Included/Excluded studies

This table represents all results of the systematic search and the included or excluded studies in addition to studies focusing on breast cancer microbiome but without mentioning breast cancer subtypes.

Title	Exclude/include	Type of samples
The Microbiota of Breast Tissue and Its Association with Breast Cancer	Excluded	no subtypes
Gut microbiota and short-chain fatty acid alterations in cachectic cancer patients	Excluded	different

Acupuncture ameliorates breast cancer-related fatigue by regulating the gut microbiota-gut-brain axis	Excluded	different
Human breast microbiome correlates with prognostic features and immunological signatures in breast cancer	Excluded	Data not available
Tissue Microbiome Associated With Human Diseases by Whole Transcriptome Sequencing and 16S Metagenomics	Excluded	Review
Variations in the Gut Microbiota in Breast Cancer Occurrence and Bone Metastasis	Excluded	different
Interactions between the breast tissue microbiota and host gene regulation in nonpuerperal mastitis	Excluded	no subtypes
Aucubin Exerts Anticancer Activity in Breast Cancer and Regulates Intestinal Microbiota	Excluded	different
Intestinal Microbiota in Postmenopausal Breast Cancer Patients and Controls	Excluded	different
Breast microbiome associations with breast tumor characteristics and neoadjuvant chemotherapy: A case-control study	included	included
Chemotherapy-associated oral microbiome changes in breast cancer patients	Excluded	different
Analysis of Gut Microbiota in Patients with Breast Cancer and Benign Breast Lesions	Excluded	different
Breast tissue, oral and urinary microbiomes in breast cancer	Excluded	Data not available
Obesity Modulates the Gut Microbiome in Triple-Negative Breast Cancer	Excluded	different
Gut microbiome associations with breast cancer risk factors and tumor characteristics: a pilot study	Excluded	different
16S full-length gene sequencing analysis of intestinal flora in breast cancer patients in Hainan Province	Excluded	different
Postmenopausal breast cancer and oestrogen associations with the IgA-coated and IgA-noncoated faecal microbiota	Excluded	no subtypes
Exploring breast tissue microbial composition and the association with breast cancer risk factors	Excluded	no subtypes
L-norvaline affects the proliferation of breast cancer cells based on the microbiome and metabolome analysis	Excluded	no subtypes
PCR Characterization of Microbiota on Contracted and Non-Contracted Breast Capsules	Excluded	no subtypes
Gut microbiome, body weight, and mammographic breast density in healthy postmenopausal women	Excluded	different
Association between Gut Microbiota and Breast Cancer: Diet as a Potential Modulating Factor	Excluded	different
Integrating current analyses of the breast cancer microbiome	Excluded	meta
Predictive and Preventive Potential of Preoperative Gut Microbiota in Chronic Postoperative Pain in Breast Cancer Survivors	Excluded	different
Naso-oropharyngeal microbiome from breast cancer patients diagnosed with COVID-19	Excluded	different
Composition and Functional Potential of the Human Mammary Microbiota Prior to and Following Breast Tumor Diagnosis	Excluded	different
Microbiota of human breast tissue	Excluded	no subtypes
The oral microbiome and breast cancer and nonmalignant breast disease, and its relationship with the fecal microbiome in the Ghana Breast Health Study	Excluded	different
Mammographic breast density and its association with urinary estrogens and the fecal microbiota in postmenopausal women	Excluded	different
Microbiome composition indicate dysbiosis and lower richness in tumor breast tissues compared to healthy adjacent paired tissue, within the same women	included	included
Breast Cancer Survivors and Healthy Women: Could Gut Microbiota Make a Difference?-"BiotaCancerSurvivors": A Case-Control Study	Excluded	different
Faecal Microbiota Composition Varies between Patients with Breast Cancer and	Excluded	different

Healthy Women: A Comparative Case-Control Study		
The Relationship of Tumor Microbiome and Oral Bacteria and Intestinal Dysbiosis in Canine Mammary Tumor	Excluded	animal model
Identifying distinctive tissue and fecal microbial signatures and the tumor-promoting effects of deoxycholic acid on breast cancer	Excluded	meta
Depletion of gut microbiota improves the therapeutic efficacy of cancer nanomedicine	Excluded	different
Breast cancer: the first comparative evaluation of oncobiome composition between males and females	Excluded	no subtypes
Effect of gastrointestinal microbiome and its diversity on the expression of tumor-infiltrating lymphocytes in breast cancer	Excluded	different
Elevated bile acid metabolism and microbiome are associated with suppressed cell proliferation and better survival in breast cancer	Excluded	no subtypes
Neoadjuvant Chemotherapy Shifts Breast Tumor Microbiota Populations to Regulate Drug Responsiveness and the Development of Metastasis	Excluded	Data not available
The Microbiome of Aseptically Collected Human Breast Tissue in Benign and Malignant Disease	Excluded	no subtypes
Potential risk of tamoxifen: gut microbiota and inflammation in mice with breast cancer	Excluded	animal model
Investigating the oral microbiome in retrospective and prospective cases of prostate, colon, and breast cancer	Excluded	different
Impact of gut permeability on the breast microbiome using a non-human primate model	Excluded	animal model
Characterization of the microbiome of nipple aspirate fluid of breast cancer survivors	Excluded	different
Comparison of the Gut Microbiota in Patients with Benign and Malignant Breast Tumors: A Pilot Study	Excluded	different
Fecal Akkermansia muciniphila Is Associated with Body Composition and Microbiota Diversity in Overweight and Obese Women with Breast Cancer Participating in a Presurgical Weight Loss Trial	Excluded	different
Antibiotic treatment targeting gram negative bacteria prevents neratinib-induced diarrhea in rats	Excluded	irrelevant
Comparative analysis of racial differences in breast tumor microbiome	included	included
The Association Between Breast Density and Gut Microbiota Composition at 2 Years Post-Menarche: A Cross-Sectional Study of Adolescents in Santiago, Chile	Excluded	different
Relationships of sleep disturbance, intestinal microbiota, and postoperative pain in breast cancer patients: a prospective observational study	Excluded	different
Characterization of human breast tissue microbiota from core needle biopsies through the analysis of multi hypervariable 16S-rRNA gene regions	Excluded	irrelevant
Potential mechanism of pyrotinib-induced diarrhea was explored by gut microbiome and ileum metabolomics	Excluded	irrelevant
Gut microbiota diversity is associated with cardiorespiratory fitness in post-primary treatment breast cancer survivors	Excluded	different
Preliminary Analysis of Gut Microbiome and Gastrointestinal Symptom Burden in Breast Cancer Patients Receiving Chemotherapy Compared to Healthy Controls	Excluded	different
Construction of model animals to explore intestinal microbiome for detection of breast cancer	Excluded	animal model
Associations of fecal microbial profiles with breast cancer and nonmalignant breast disease in the Ghana Breast Health Study	Excluded	different
Microbiome Diversity in Sputum of Nontuberculous Mycobacteria Infected Women with a History of Breast Cancer	Excluded	no subtypes
Biopsy bacterial signature can predict patient tissue malignancy	Excluded	no subtypes
Characterization of the Metabolome of Breast Tissues from Non-Hispanic Black and Non-Hispanic White Women Reveals Correlations between Microbial Dysbiosis and	Excluded	Data not available

Enhanced Lipid Metabolism Pathways in Triple-Negative Breast Tumors		
The microbiome in PTEN hamartoma tumor syndrome	Excluded	different
Gut microbial differences in breast and prostate cancer cases from two randomised controlled trials compared to matched cancer-free controls	Excluded	different
The effect of Poria cocos ethanol extract on the intestinal barrier function and intestinal microbiota in mice with breast cancer	Excluded	animal model
Gut Microbiota Profiling in Patients With HER2-Negative Metastatic Breast Cancer Receiving Metronomic Chemotherapy of Capecitabine Compared to Those Under Conventional Dosage	Excluded	different
Potential values of formalin-fixed paraffin-embedded tissues for intratumoral microbiome analysis in breast cancer	Excluded	no subtypes
Gut microbiota composition associated with alterations in cardiorespiratory fitness and psychosocial outcomes among breast cancer survivors	Excluded	different
Distinct microbial communities that differ by race, stage, or breast-tumor subtype in breast tissues of non-Hispanic Black and non-Hispanic White women	included	included
Reduction of Staphylococcus epidermidis in the mammary tumor microbiota induces antitumor immunity and decreases breast cancer aggressiveness	Excluded	animal model
Species-Level Characterization of the Microbiome in Breast Tissues with Different Malignancy and Hormone-Receptor Statuses Using Nanopore Sequencing	Excluded	Different seq, method
Associations of Circulating Estrogens and Estrogen Metabolites with Fecal and Oral Microbiome in Postmenopausal Women in the Ghana Breast Health Study	Excluded	different
The effect of fucoidan on intestinal flora and intestinal barrier function in rats with breast cancer	Excluded	animal model
Study of Microbiomes in Aseptically Collected Samples of Human Breast Tissue Using Needle Biopsy and the Potential Role of in situ Tissue Microbiomes for Promoting Malignancy	Excluded	Diff variable region
Probiotics for the Treatment of Docetaxel-Related Weight Gain of Breast Cancer Patients-A Single-Center, Randomized, Double-Blind, and Placebo-Controlled Trial	Excluded	different
Health-related quality of life is associated with fecal microbial composition in breast cancer survivors	Excluded	different
Intra-tumoral microbial community profiling and associated metabolites alterations of TNBC	Excluded	Data not available
Anti-breast Cancer Enhancement of a Polysaccharide From Spore of Ganoderma lucidum With Paclitaxel: Suppression on Tumor Metabolism With Gut Microbiota Reshaping	Excluded	irrelevant
Faecal microbiota composition is related to response to CDK4/6-inhibitors in metastatic breast cancer: A prospective cross-sectional exploratory study	Excluded	different
Associations of gut microbiome with endogenous estrogen levels in healthy postmenopausal women	Excluded	irrelevant
Physiological and cognitive changes after treatments of cyclophosphamide, methotrexate, and fluorouracil: implications of the gut microbiome and depressive-like behavior	Excluded	irrelevant
Metagenomics Analysis of Breast Microbiome Highlights the Abundance of Rothia Genus in Tumor Tissues	Excluded	Data not available
Investigation of the association between the fecal microbiota and breast cancer in postmenopausal women: a population-based case-control pilot study	Excluded	different
Changes in gut microbiota composition after 12 weeks of a home-based lifestyle intervention in breast cancer survivors during the COVID-19 lockdown	Excluded	irrelevant
Intestinal Proportion of Blautia sp. is Associated with Clinical Stage and Histoprognostic Grade in Patients with Early-Stage Breast Cancer	Excluded	different
Three phytosterols from sweet potato inhibit MCF7-xenograft-tumor growth through modulating gut microbiota homeostasis and SCFAs secretion	Excluded	cell line

Toxicity, Pharmacokinetics, and Gut Microbiome of Oral Administration of Sesterterpene MHO7 Derived from a Marine Fungus	Excluded	irrelevant
Annexin-A1 deficiency attenuates stress-induced tumor growth via fatty acid metabolism in mice: an Integrated multiple omics analysis on the stress- microbiome-metabolite-epigenetic-oncology (SMMEO) axis	Excluded	animal model
Comparison of gut microbiome composition in colonic biopsies, endoscopically-collected and at-home-collected stool samples	Excluded	different
Breast cancer patients from the Midwest region of the United States have reduced levels of short-chain fatty acid-producing gut bacteria	Excluded	different
Analysis of Gut Microbiome Using Explainable Machine Learning Predicts Risk of Diarrhea Associated With Tyrosine Kinase Inhibitor Neratinib: A Pilot Study	Excluded	different
Associations of the fecal microbiome with urinary estrogens and estrogen metabolites in postmenopausal women	Excluded	different
Basolateral Secretion from Caco-2 Cells Pretreated with Fecal Waters from Breast Cancer Patients Affects MCF7 Cell Viability	Excluded	cell line
Effects of Branched-Chain Fatty Acids Derived from Yak Ghee on Lipid Metabolism and the Gut Microbiota in Normal-Fat Diet-Fed Mice	Excluded	animal model
Fecal microbial determinants of fecal and systemic estrogens and estrogen metabolites: a cross-sectional study	Excluded	different
Antitumor Activity of Extract From the Sporoderm-Breaking Spore of Ganoderma lucidum: Restoration on Exhausted Cytotoxic T Cell With Gut Microbiota Remodeling	Excluded	different
Production of enterodiol from defatted flaxseeds through biotransformation by human intestinal bacteria	Excluded	different

Supplementary Table 2: Differentially Abundant Taxonomical Levels in TNBC and Non-TNBC

This table represents the abundant taxonomical levels in TNBC and Non-TNBC according to ANCOM-BC.

	TNBC	NTNBC
Class	Acidobacteria_Gp7, Hydrogenophilalia, Spartobacteria	
Order	Acidaminococcales, Anaerolineales, Deinococcales, Deltaproteobacteriaspp, Gammaproteobacteria_incertae_sedis, Gp6, Gp7, Hydrogenophilales, Selenomonadales, Spartobacteria spp, Streptosporangiales	
Family	Acidaminococcaceae, Acidibacter, Aerococcaceae, Anaerolineaceae, Azospirillaceae, Bacillaceae_2, Clostridiales spp, Deinococcaceae, Deltaproteobacteria spp, Gp6 spp, Gp7 spp, Helicobacteraceae, Hydrogenophilaceae, Kribbellaceae, Lactobacillales spp, Selenomonadaceae, Solirubrobacterales spp, Spartobacteria spp, Thermoleophilia spp	Arenimicrobiaceae
Genus	Acidibacter, Aerococcus, Amnibacterium, Anaerobutyricum, Anaerolineaceaespp, Anaerotignum, Azospirillum, Clostridialesspp, Clostridium_XVIII, Deinococcus, Deltaproteobacteriaspp, Enterocloster, Glutamicibacter, Gp6spp, Gp7spp, Helicobacter, Hydrogenophilus, Kibdelosporangium, Kribbella, Lachnospiraceaespp, Lactobacillalesspp, Lentilactobacillus, Levilactobacillus, Ligilactobacillus, Limosilactobacillus, Lysobacter, Mediterraneibacter, Neglecta, Oceanospirillaceaespp, Parvimonas, Propionispira,	Alteromonas, Actinobacteriaspp, Alteromonas, Chromatialesspp, Gammaproteobacteriaspp, Ruminococcus, Bacteriaspp, Paracoccus.

	Ruminococcaceaespp, Schlegelella, Skermanella, Solirubrobacteralesspp, Spartobacteriaspp, Sphingorhabdus, Tepidimonas, Terrisporobacter, Thermoleophiliaspp, Vulcaniibacterium, Bacteriaspp, Dialister, Solirubrobacter, Gaiella, Bacteroides, Clostridium_sensu_stricto, Aureimonas, Comamonadaceaespp, Thermus, Betaproteobacteriaspp, Intestinimonas, Bacteroidetesspp, Streptococcus, Vibrio, Blautia, Prevotella, Rothia, Faecalibaculum, Gammaproteobacteriaspp, Microbacteriaceaespp, Rubrobacter, Alistipes, Bacillalesspp, Bacillaceae_1spp.	

Supplementary Table 3: Association Analysis Pathway Results

This table represents the significant pathways in TNBC compared to Non-TNBC according to the association analysis of MicrobiomeAnalyst

Pathway	Size	Hits	Statistic Q	Expected Q	Pval	Holm p	FDR
Taurine and hypotaurine metabolism	10	10	4.02327	0.502513	0.000267	0.031829	0.018204
Steroid biosynthesis	5	5	3.759087	0.502513	0.000548	0.064627	0.018204
Monobactam biosynthesis	6	6	3.170049	0.502513	0.00063	0.073699	0.018204
Alanine, aspartate and glutamate metabolism	16	16	2.871927	0.502513	0.000798	0.092564	0.018204
Arginine biosynthesis	19	19	2.935406	0.502513	0.00084	0.096631	0.018204
Sesquiterpenoid and triterpenoid biosynthesis	4	4	3.599766	0.502513	0.000918	0.104633	0.018204
Ascorbate and aldarate metabolism	26	26	2.899564	0.502513	0.001452	0.16406	0.024143
Carbon fixation pathways in prokaryotes	26	26	2.223028	0.502513	0.001623	0.181782	0.024143
Cysteine and methionine metabolism	38	38	2.256889	0.502513	0.002166	0.240421	0.028639
Amino sugar and nucleotide sugar metabolism	63	63	1.966124	0.502513	0.002921	0.321326	0.03165
Phenylalanine, tyrosine and tryptophan biosynthesis	19	19	2.142892	0.502513	0.002926	0.321326	0.03165
Methane metabolism	51	51	1.503561	0.502513	0.004129	0.445964	0.040949
Valine, leucine and isoleucine biosynthesis	3	3	3.485476	0.502513	0.005906	0.631945	0.054063
O-Antigen nucleotide sugar biosynthesis	28	28	1.872328	0.502513	0.007271	0.770705	0.061802
Linoleic acid metabolism	2	2	2.651785	0.502513	0.009286	0.974999	0.073667
Caprolactam degradation	6	6	2.102909	0.502513	0.010165	1	0.075603
Glycosaminoglycan degradation	8	8	1.938643	0.502513	0.011177	1	0.07599
Aminobenzoate degradation	14	14	2.073594	0.502513	0.011494	1	0.07599
Pentose and glucuronate interconversions	37	37	1.821239	0.502513	0.012841	1	0.080427
Biotin metabolism	7	7	2.16113	0.502513	0.013608	1	0.080969
C5-Branched dibasic acid metabolism	9	9	2.162955	0.502513	0.015789	1	0.082783
Citrate cycle (TCA cycle)	12	12	1.711686	0.502513	0.017173	1	0.082783
Metabolism of xenobiotics by cytochrome P450	3	3	2.077074	0.502513	0.017504	1	0.082783
Arachidonic acid metabolism	2	2	2.381445	0.502513	0.017748	1	0.082783

Ethylbenzene degradation	3	3	2.030649	0.502513	0.017844	1	0.082783
Drug metabolism - cytochrome P450	4	4	1.989041	0.502513	0.01836	1	0.082783
Glycosphingolipid biosynthesis - globo and isoglobo series	2	2	2.297114	0.502513	0.018783	1	0.082783
Oxidative phosphorylation	9	9	1.746767	0.502513	0.031059	1	0.131999
Valine, leucine and isoleucine degradation	19	19	1.435662	0.502513	0.032238	1	0.132287
Glycerolipid metabolism	13	13	1.452915	0.502513	0.037703	1	0.147178
Selenocompound metabolism	7	7	1.537587	0.502513	0.03834	1	0.147178
Tropane, piperidine and pyridine alkaloid biosynthesis	5	5	1.310039	0.502513	0.045016	1	0.167405
Folate biosynthesis	14	14	1.245669	0.502513	0.050544	1	0.182264
Pyruvate metabolism	29	29	1.254467	0.502513	0.052475	1	0.183663
Penicillin and cephalosporin biosynthesis	4	4	1.320843	0.502513	0.054911	1	0.186696
Lipoic acid metabolism	8	8	1.339006	0.502513	0.058839	1	0.194496
Ether lipid metabolism	4	4	1.352628	0.502513	0.071772	1	0.224367
Lysine biosynthesis	29	29	1.001113	0.502513	0.071779	1	0.224367
Pentose phosphate pathway	28	28	1.091519	0.502513	0.07456	1	0.224367
Novobiocin biosynthesis	3	3	1.299557	0.502513	0.075417	1	0.224367
Starch and sucrose metabolism	40	40	0.971484	0.502513	0.078751	1	0.22857
Sphingolipid metabolism	9	9	1.14001	0.502513	0.08698	1	0.243852
Lipopolysaccharide biosynthesis	20	20	1.145748	0.502513	0.088115	1	0.243852
Teichoic acid biosynthesis	2	2	1.395513	0.502513	0.092048	1	0.248949
Inositol phosphate metabolism	14	14	1.001922	0.502513	0.098116	1	0.259461
Glycine, serine and threonine metabolism	38	38	0.940863	0.502513	0.104699	1	0.2629
Vitamin B6 metabolism	7	7	1.119022	0.502513	0.105714	1	0.2629
Atrazine degradation	6	6	1.064143	0.502513	0.106044	1	0.2629
Steroid hormone biosynthesis	3	3	1.093317	0.502513	0.11136	1	0.267723
Histidine metabolism	13	13	0.954917	0.502513	0.1146	1	0.267723
Riboflavin metabolism	14	14	0.912901	0.502513	0.114738	1	0.267723
Tetracycline biosynthesis	3	3	1.147129	0.502513	0.118618	1	0.271453
Pinene, camphor and geraniol degradation	3	3	1.051379	0.502513	0.125929	1	0.282747
Phenylpropanoid biosynthesis	3	3	0.974977	0.502513	0.134179	1	0.290772
Thiamine metabolism	14	14	0.911275	0.502513	0.134536	1	0.290772
Glycerophospholipid metabolism	11	11	0.944818	0.502513	0.136834	1	0.290772
Sulfur metabolism	15	15	0.886107	0.502513	0.14143	1	0.293419
Staurosporine biosynthesis	2	2	0.97085	0.502513	0.144798	1	0.293419
Carbon fixation in photosynthetic organisms	10	10	0.960088	0.502513	0.146078	1	0.293419
Biosynthesis of type II polyketide backbone	3	3	0.942577	0.502513	0.147942	1	0.293419
Biosynthesis of various other secondary metabolites	4	4	0.882167	0.502513	0.174551	1	0.329437
Glycolysis / Gluconeogenesis	27	27	0.767702	0.502513	0.17505	1	0.329437
Dioxin degradation	9	9	0.847495	0.502513	0.17515	1	0.329437
alpha-Linolenic acid metabolism	2	2	0.884403	0.502513	0.177449	1	0.329437
Fructose and mannose metabolism	41	41	0.740275	0.502513	0.179945	1	0.329437

Glutathione metabolism	12	12	0.783902	0.502513	0.184331	1	0.332355
Ubiquinone and other terpenoid-quinone biosynthesis	23	23	0.717677	0.502513	0.199794	1	0.353206
Other glycan degradation	3	3	0.798024	0.502513	0.201832	1	0.353206
Benzoate degradation	45	45	0.715418	0.502513	0.21114	1	0.359124
beta-Alanine metabolism	18	18	0.716077	0.502513	0.211703	1	0.359124
Photosynthesis	5	5	0.733626	0.502513	0.215178	1	0.359124
Carotenoid biosynthesis	12	12	0.704432	0.502513	0.217285	1	0.359124
Tryptophan metabolism	20	20	0.693534	0.502513	0.228851	1	0.369817
Chloroalkane and chloroalkene degradation	7	7	0.703351	0.502513	0.22997	1	0.369817
Pantothenate and CoA biosynthesis	14	14	0.658678	0.502513	0.238187	1	0.376678
Propanoate metabolism	32	32	0.661014	0.502513	0.240783	1	0.376678
Isoquinoline alkaloid biosynthesis	6	6	0.666488	0.502513	0.243733	1	0.376678
Phenylalanine metabolism	29	29	0.647743	0.502513	0.249555	1	0.380731
Drug metabolism - other enzymes	15	15	0.63582	0.502513	0.257241	1	0.38749
Pyrimidine metabolism	35	35	0.616297	0.502513	0.267245	1	0.396417
Polycyclic aromatic hydrocarbon degradation	10	10	0.640907	0.502513	0.26983	1	0.396417
D-Amino acid metabolism	15	15	0.605169	0.502513	0.274086	1	0.397759
Porphyrin metabolism	37	37	0.575142	0.502513	0.303844	1	0.435631
Nicotinate and nicotinamide metabolism	15	15	0.566378	0.502513	0.309523	1	0.438491
Various types of N-glycan biosynthesis	3	3	0.495111	0.502513	0.330378	1	0.46253
Cyanoamino acid metabolism	6	6	0.49815	0.502513	0.337913	1	0.467351
Xylene degradation	20	20	0.468415	0.502513	0.341677	1	0.467351
Lysine degradation	13	13	0.520264	0.502513	0.348163	1	0.470811
Purine metabolism	43	43	0.5303	0.502513	0.354178	1	0.473564
Butanoate metabolism	31	31	0.521783	0.502513	0.363661	1	0.480841
Primary bile acid biosynthesis	2	2	0.477631	0.502513	0.375433	1	0.489879
Arginine and proline metabolism	44	44	0.498599	0.502513	0.392901	1	0.489879
Fatty acid degradation	8	8	0.465104	0.502513	0.393186	1	0.489879
Chlorocyclohexane and chlorobenzene degradation	15	15	0.472046	0.502513	0.395604	1	0.489879
Styrene degradation	9	9	0.394414	0.502513	0.399664	1	0.489879
Naphthalene degradation	8	8	0.468695	0.502513	0.4018	1	0.489879
Nitrotoluene degradation	4	4	0.454617	0.502513	0.402336	1	0.489879
Biosynthesis of unsaturated fatty acids	3	3	0.376043	0.502513	0.403856	1	0.489879
Tyrosine metabolism	28	28	0.486857	0.502513	0.409427	1	0.489879
Glyoxylate and dicarboxylate metabolism	36	36	0.460918	0.502513	0.41336	1	0.489879
Toluene degradation	17	17	0.472423	0.502513	0.41578	1	0.489879
Terpenoid backbone biosynthesis	12	12	0.360587	0.502513	0.445179	1	0.519375
Fluorobenzoate degradation	9	9	0.310807	0.502513	0.477943	1	0.547009
Peptidoglycan biosynthesis	18	18	0.337328	0.502513	0.478059	1	0.547009
One carbon pool by folate	7	7	0.326743	0.502513	0.502702	1	0.563265
Photosynthesis - antenna proteins	3	3	0.348967	0.502513	0.503197	1	0.563265

N-Glycan biosynthesis	6	6	0.354132	0.502513	0.508084	1	0.563265
Phosphonate and phosphinate metabolism	9	9	0.33496	0.502513	0.511198	1	0.563265
Fatty acid biosynthesis	5	5	0.206426	0.502513	0.638946	1	0.697565
Biosynthesis of various antibiotics	4	4	0.269732	0.502513	0.647901	1	0.700912
Betalain biosynthesis	2	2	0.124309	0.502513	0.659414	1	0.70694
Caffeine metabolism	2	2	0.141107	0.502513	0.666123	1	0.707755
Nitrogen metabolism	15	15	0.242876	0.502513	0.722478	1	0.76084
Galactose metabolism	26	26	0.212283	0.502513	0.751196	1	0.784143
Streptomycin biosynthesis	4	4	0.083852	0.502513	0.763962	1	0.790534
Steroid degradation	7	7	0.063287	0.502513	0.850343	1	0.872335
Biosynthesis of 12-, 14- and 16-membered macrolides	2	2	0.013429	0.502513	0.870944	1	0.885832
Biosynthesis of enediyne antibiotics	2	2	0.018377	0.502513	0.930365	1	0.938249
Biosynthesis of type II polyketide products	5	5	0.023505	0.502513	0.968549	1	0.968549

Supplementary Table 4: PRISMA checklist for Meta-analysis

PRISMA 2020 Checklist

Section and Topic	Item #	Checklist item	Location where item is reported
**TITLE**			
Title	1	Identify the report as a systematic review.	Title (reported as meta-analysis)
**ABSTRACT**			
Abstract	2	See the PRISMA 2020 for Abstracts checklist.	Abstract
**INTRODUCTION**			
Rationale	3	Describe the rationale for the review in the context of existing knowledge.	Section 1.0 Introduction and<br>Section 3.0 Discussion
Objectives	4	Provide an explicit statement of the objective(s) or question(s) the review addresses.	Section 1.0 Introduction
**METHODS**			
Eligibility criteria	5	Specify the inclusion and exclusion criteria for the review and how studies were grouped for the syntheses.	Figure 1, Section 2.1 Systematic Search and<br>Section 4.2 Data Pre-processing
Information sources	6	Specify all databases, registers, websites, organisations, reference lists and other sources searched or consulted to identify studies. Specify the date when each source was last searched or consulted.	Section 2.1 Systematic Search
Search strategy	7	Present the full search strategies for all databases, registers and websites, including any filters and limits used.	Section 2.1 Systematic Search and Supplementary Data 1
Selection process	8	Specify the methods used to decide whether a study met the inclusion criteria of the review, including how many reviewers screened each record and each report retrieved, whether they worked independently, and if applicable, details of automation tools used in the process.	Figure 1 and<br>Section 2.1 Systematic Search
Data collection process	9	Specify the methods used to collect data from reports, including how many reviewers collected data from each report, whether they worked independently, any processes for obtaining or confirming data from study investigators, and if applicable, details of automation tools used in the process.	Section 2.2 Data Pre-processing
Data items	10a	List and define all outcomes for which data were sought. Specify whether all results that were compatible with each outcome domain in each study were sought (e.g. for all measures, time points, analyses), and if not, the methods used to decide which results to collect.	Section 2.2 Data Pre-processing and Supplementary Table 1
	10b	List and define all other variables for which data were sought (e.g. participant and intervention characteristics, funding sources). Describe any assumptions made about any missing or unclear information.	Not applicable
Study risk of bias assessment	11	Specify the methods used to assess risk of bias in the included studies, including details of the tool(s) used, how many reviewers assessed each study and whether they worked independently, and if applicable, details of automation tools used in the process.	Section 2.2 Data Pre-processing and<br>Figure 1
Effect measures	12	Specify for each outcome the effect measure(s) (e.g. risk ratio, mean difference) used in the synthesis or presentation of results.	Section 2.4 Biomarkers Detection through Meta-analysis
Synthesis methods	13a	Describe the processes used to decide which studies were eligible for each synthesis (e.g. tabulating the study intervention characteristics and comparing against the planned groups for each synthesis (item #5)).	Section 2.1 Systematic Search and<br>Figure 1

Section and Topic	Item #	Checklist item	Location where item is reported
	13b	Describe any methods required to prepare the data for presentation or synthesis, such as handling of missing summary statistics, or data conversions.	Section 2.2 Data Pre-processing and 2.3 Diversity analysis
	13c	Describe any methods used to tabulate or visually display results of individual studies and syntheses.	Section 2.2 Data Pre-processing
	13d	Describe any methods used to synthesize results and provide a rationale for the choice(s). If meta-analysis was performed, describe the model(s), method(s) to identify the presence and extent of statistical heterogeneity, and software package(s) used.	Section 2.2 Data Pre-processing and Supplementary Table 1
	13e	Describe any methods used to explore possible causes of heterogeneity among study results (e.g. subgroup analysis, meta-regression).	Section 2.2 Data Pre-processing
	13f	Describe any sensitivity analyses conducted to assess robustness of the synthesized results.	Section 2.2 Data Pre-processing and Figure 1
Reporting bias assessment	14	Describe any methods used to assess risk of bias due to missing results in a synthesis (arising from reporting biases).	Section 2.2 Data Pre-processing and Figure 1
Certainty assessment	15	Describe any methods used to assess certainty (or confidence) in the body of evidence for an outcome.	Section 2.4 Biomarkers Detection through Meta-analysis
**RESULTS**			
Study selection	16a	Describe the results of the search and selection process, from the number of records identified in the search to the number of studies included in the review, ideally using a flow diagram.	Figure 1 and Section 3.1
	16b	Cite studies that might appear to meet the inclusion criteria, but which were excluded, and explain why they were excluded.	Supplementary Data 1
Study characteristics	17	Cite each included study and present its characteristics.	Figure 1 and Table 1 and Section 3.1
Risk of bias in studies	18	Present assessments of risk of bias for each included study.	Section 3.1
Results of individual studies	19	For all outcomes, present, for each study: (a) summary statistics for each group (where appropriate) and (b) an effect estimate and its precision (e.g. confidence/credible interval), ideally using structured tables or plots.	Table 1
Results of syntheses	20a	For each synthesis, briefly summarise the characteristics and risk of bias among contributing studies.	Table 1 and Section 3.2
	20b	Present results of all statistical syntheses conducted. If meta-analysis was done, present for each the summary estimate and its precision (e.g. confidence/credible interval) and measures of statistical heterogeneity. If comparing groups, describe the direction of the effect.	Supplementary Data 1 Section 3.5
	20c	Present results of all investigations of possible causes of heterogeneity among study results.	Figure 1 and Section 3.1 and Supplementary Data 1
	20d	Present results of all sensitivity analyses conducted to assess the robustness of the synthesized results.	Figure 1 and Section 3.1 and 3.2
Reporting biases	21	Present assessments of risk of bias due to missing results (arising from reporting biases) for each synthesis assessed.	Figure 1 and Section 3.1
Certainty of evidence	22	Present assessments of certainty (or confidence) in the body of evidence for each outcome assessed.	Figure 1 and Section 3.1
**DISCUSSION**			

Section and Topic	Item #	Checklist item	Location where item is reported
and Discussion	23a	Provide a general interpretation of the results in the context of other evidence.	Section 4.0 Discussion and Section 1.0 Introduction
	23b	Discuss any limitations of the evidence included in the review.	Section 4.0 Discussion
	23c	Discuss any limitations of the review processes used.	Section 4.0 Discussion
	23d	Discuss implications of the results for practice, policy, and future research.	Section 4.0 Discussion and Section 1.0 Introduction
**OTHER INFORMATION**			
Registration and protocol	24a	Provide registration information for the review, including register name and registration number, or state that the review was not registered.	Not applicable
	24b	Indicate where the review protocol can be accessed, or state that a protocol was not prepared.	Section 6.3 Data availability
	24c	Describe and explain any amendments to information provided at registration or in the protocol.	Not applicable
Support	25	Describe sources of financial or non-financial support for the review, and the role of the funders or sponsors in the review.	not applicable
Competing interests	26	Declare any competing interests of review authors.	Section 6.4 Competing interests
Availability of data, code and other materials	27	Report which of the following are publicly available and where they can be found: template data collection forms; data extracted from included studies; data used for all analyses; analytic code; any other materials used in the review.	Table 1 and Section 6.3 Data availability and Section 6.3 Code availability

From: Page MJ, McKenzie JE, Bossuyt PM, Boutron I, Hoffmann TC, Mulrow CD, et al. The PRISMA 2020 statement: an updated guideline for reporting systematic reviews. BMJ 2021;372:n71. doi: 10.1136/bmj.n71. This work is licensed under CC BY 4.0. To view a copy of this license, visit https://creativecommons.org/licenses/by/4.0/

Section and Topic	Item #	Checklist item	Reported (Yes/No)
**TITLE**			
Title	1	Identify the report as a systematic review.	Yes (reported as meta-analysis)
**BACKGROUND**			
Objectives	2	Provide an explicit statement of the main objective(s) or question(s) the review addresses.	Yes
**METHODS**			
Eligibility criteria	3	Specify the inclusion and exclusion criteria for the review.	Yes
Information sources	4	Specify the information sources (e.g. databases, registers) used to identify studies and the date when each was last searched.	Yes
Risk of bias	5	Specify the methods used to assess risk of bias in the included studies.	Yes
Synthesis of results	6	Specify the methods used to present and synthesise results.	Yes
**RESULTS**			
Included studies	7	Give the total number of included studies and participants and summarise relevant characteristics of studies.	Yes
Synthesis of results	8	Present results for main outcomes, preferably indicating the number of included studies and participants for each. If meta-analysis was done, report the summary estimate and confidence/credible interval. If comparing groups, indicate the direction of the effect (i.e. which group is favoured).	Yes
**DISCUSSION**			
Limitations of evidence	9	Provide a brief summary of the limitations of the evidence included in the review (e.g. study risk of bias, inconsistency and imprecision).	No
Interpretation	10	Provide a general interpretation of the results and important implications.	Yes
**OTHER**			
Funding	11	Specify the primary source of funding for the review.	Yes
Registration	12	Provide the register name and registration number.	No

PRISMA 2020 for Abstracts Checklist

From: Page MJ, McKenzie JE, Bossuyt PM, Boutron I, Hoffmann TC, Mulrow CD, et al. The PRISMA 2020 statement: an updated guideline for reporting systematic reviews. BMJ 2021;372:n71. doi: 10.1136/bmj.n71. This work is licensed under CC BY 4.0. To view a copy of this license, visit https://creativecommons.org/licenses/by/4.0/
